# Collaborating in Isolation: Assessing the Effects of the Covid-19 Pandemic on Patterns of Collaborative Behavior Among Working Musicians

**DOI:** 10.3389/fpsyg.2021.674246

**Published:** 2021-07-19

**Authors:** Noah R. Fram, Visda Goudarzi, Hiroko Terasawa, Jonathan Berger

**Affiliations:** ^1^Center for Computer Research in Music and Acoustics, Music Department, Stanford University, Stanford, CA, United States; ^2^Department of Audio Arts and Acoustics, Columbia College Chicago, Chicago, IL, United States; ^3^Faculty of Library, Information and Media Science, University of Tsukuba, Tsukuba, Japan

**Keywords:** social media, musical collaboration, technology use, COVID-19 pandemic, musical genre

## Abstract

The Covid-19 pandemic severely limited collaboration among musicians in rehearsal and ensemble performance, and demanded radical shifts in collaborative practices. Understanding the nature of these changes in music creators' patterns of collaboration, as well as how musicians shifted prioritizations and adapted their use of the available technologies, can offer invaluable insights into the resilience and importance of different aspects of musical collaboration. In addition, assessing changes in the collaboration networks among music creators can improve the current understanding of genre and style formation and evolution. We used an internet survey distributed to music creators, including performers, composers, producers, and engineers, all active before and during the pandemic, to assess their perceptions of how their music, collaborative practice, and use of technology were impacted by shelter-in-place orders associated with Covid-19, as well as how they adapted over the course of the pandemic. This survey was followed by Zoom interviews with a subset of participants. Along with confirming previous results showing increased reliance on nostalgia for musical inspiration, we found that participants' collaborative behaviors were surprisingly resilient to pandemic-related changes. In addition, participant responses appeared to be driven by a relatively small number of underlying factors, representing approaches to musical collaboration such as *musical extroversion* or *musical introversion*, inspiration clusters such as *activist musicking*, and style or genre clusters.

## 1. Introduction

Making music with others, or collaborative music-making, is a core aspect of human musicality (Small, 1998). Traditionally, collaborative music making has predominantly been face-to-face, synchronous and interactive, with reliance on recording and production technologies for dissemination and archiving. The emergence of electroacoustic synthesis and processing, along with interactive digital systems, has brought new technologies to the forefront of music practices, and new genres such as electronic music and live coding have changed the shape of musicking and collaborative music creation. Creative usage of the Internet, of mobile devices, and of embedded technologies for ubiquitous musical activities has also changed the relation between bodily expression, or the physical actions associated with making music, and music performance, particularly in domains such as electronic music (Peters et al., 2012) and responsive music technologies (Einarsson and Ziemke, 2017). With the rise of the “Internet of musical things,” augmented and immersive concerts experiences, remote rehearsals, audience participation, music e-learning, and smart studio production have been taking shape (Turchet et al., 2018).

This study investigates how musicians proactively adapted internet-based technologies to perform and produce music, and to maintain (or retain) inspiration to create during the COVID-19 pandemic. Specifically, we are interested in two questions: What aspects of musical collaboration do music-makers strive to preserve and maintain? What social, cultural, and technological affordances do they rely on to do so? The Covid-19 pandemic offers a unique opportunity to search for patterns in the usage of these new technological capacities, especially in the community of professional and semi-professional music-makers such as performers, songwriters, composers, arrangers, producers, and engineers; this is the community of music-makers we refer to with the phrase “working musicians.”

Since it represents a significant extrinsic shock to the normal operation of musical interaction, community-building, and collaboration that could only be surmounted by relying on the emerging technology for music production, the pandemic can be treated as a natural experiment. In particular, any patterns in how music-makers turned to new technological tools to resolve the pandemic's drastic effects on traditional forms of musical collaboration and interaction would reflect the priorities of music-makers and the shape of the structures they work within. It stands to reason that music-makers would preferentially seek ways to continue in their most comfortable form of musical practice, and that they would do so within the constraints of their normal musical genres and collaborative networks. The present study aimed to assess these claims.

It has already been established that the pandemic has interfered with the normal forms of music-making, especially those forms that involve group interactions, in a variety of ways. Traditional music configurations such as orchestras, chamber ensembles, bands, etc., have been most affected due to the curtailment of coordinated simultaneous synchronous music performance. Taylor et al. (2020) see this as a crisis of spatial materiality. During a time of lockdown and social distancing, spaces of music production (e.g., rehearsal spaces, studios) and consumption (e.g., venues, nightclubs) have suddenly become unfit for their intended purposes. In their place, alternative approaches to music-making that prioritize accessibility and the use of technologies for remote musical interactions, such as the ubiquitous music making or ubimus community, have thrived (Keller et al., 2020). Some musicians have also adapted their use of technology, or have introduced new technological aspects to their creative and collaborative practice.

Technology may also be co-evolving to better support those creative needs. Collaborative networks of musicians in social media are not new and have precedents prior to the pandemic. Teitelbaum et al. (2008) studied the community structures and the driving forces behind the growth of such networks. They found a strong correlation between clusters of artists and musical genres. They detected two different kinds of communities: small structures related to music bands and geographic zones, and much bigger communities built upon collaborative clusters with a high number of participants related through the period the artists were active. In addition, technology has been used extensively in music pedagogy for a variety of purposes, with the potential for significantly more integration in the near future (Brown, 2007; King and Himonides, 2016; Waddell and Williamon, 2019).

Collaborative creativity in music making does not have to be bound to a physical space. Sawyer perceives improvisation, collaboration and emergence as defining characteristics of group creativity (Sawyer, 2006). Entering the state of group flow is another essential factor in collaborative music making. Mutual engagement occurs when people creatively spark together and enter a state of group flow which could be possible by providing a shared annotation mechanism (Bryan-Kinns and Hamilton, 2012). Klügel et al. (2014) distinguish among different motivators for collaborative music-making, highlighting group flow and awareness, endogenous or function-driven, and exogenous or self-justifying sources of motivation. Furthermore, new collaborative tools such as the shared virtual environment (SVE) LeMo allow people to compose music collaboratively using virtual working spaces (Men and Bryan-Kinns, 2019). Another form of music that has been taking shape in recent years is live coding. Collaborative live coding tools allow musicians to compose and perform together from disgraced geographical locations (Kirkbride, 2017). For example, CodeBank utilises public and private working environments within musical performance over a network (Keller et al., 2020). However, there is emerging evidence that music-makers have generally resorted to techniques such as asynchronous recording during the pandemic, rather than turn to unfamiliar frameworks enabling network-based musical collaboration (Onderdijk et al., 2021a).

During times of personal or collective crisis – such as a global pandemic –it is common for people to turn to music, due to its connective and unifying purposes (Bodner and Gilboa, 2009). This has manifested in a variety of ways during the Covid-19 pandemic. Some research points to light jazz or classical music, Native American, Celtic and Indian stringed-instruments, drums, and flutes, rain, wind, and other nature sounds as being effective stress reducers (Porshi, 2020). In Italy, people isolated by the pandemic sought a sense of community by standing on apartment balconies, singing “Bella Ciao” (Horowitz, 2020). The Rotterdam Philharmonic Orchestra filmed themselves playing Ludwig von Beethoven's “Ode to Joy” along with a metronome track and then assembled a compilation video of their asynchronously recorded performance (Roberts, 2020).

Many people draw additional comfort from reminiscences or other connections to their past. These feelings of nostalgia, especially those associated with emotional memories, are often stimulated by music. Analyzing data from almost 17 trillion plays of songs on Spotify in six European countries, Yeung provides evidence to suggest that the lockdown has significantly changed music consumption in terms of listeners' feelings of nostalgia (Yeung, 2020). In addition, Gibbs and Egermann showed that this nostalgic music listening does, indeed, have the theorized positive impact on emotional wellbeing, although this is mediated by the emotion regulation strategy used (Gibbs and Egermann, 2021). Nostalgia tends to respond to the drastic and lasting change caused by the lockdown, rather than to fluctuations in the viral infection. It reminds people of the good old days.

Regardless of these often inspiring instances of musical connectivity, the pandemic has had a profound negative impact on the wellbeing of musicians themselves. Cohen and Ginsborg found that professional orchestral musicians experienced a sense of loss in their personal music-making life and anxiety about the future of the music industry (Cohen and Ginsborg, 2021). Shelter-in-place has also had particularly negative effects on the elderly or other groups that have been entirely isolated by the need to slow the spread. In addition to feelings of isolation, it has been associated with increased prevalence of sadness, anxiety, irregular sleep patterns, lethargy, lack of motivation, and emotional instability for these communities, although there is evidence that these effects were ameliorated by physical activity (Spiro et al., 2021). Some musical therapists have proposed frameworks around using acoustic stimuli mainly based on soundscape compositions for influencing mood, stimulating the feeling of safety and triggering a response in a person (Hirza et al., 2020).

Livestreamed concerts have been one of the most popular cultural outlets during the COVID-19 lockdown in parts of the world. In an effort to analyze this shift in musical culture, Vandenberg et al. (2020) studied the collective consciousness, and the related feelings of social solidarity and resilience, while physical distancing recommendations were in effect by analyzing the comments during livestreamed techno concerts in the Netherlands. They found that both old and new ritual actions are used to form online communities. Parsons looks at the emergence of a variety of streaming events such as Virtual Coffee Concerts, Streaming Sundays, Musicians on Call (Parsons, 2020). In Quebec (Canada) government solicited the assistance of local music artists to capture the population's attention quickly and massively to communicate public health recommendations against the spread of COVID-19 (Lemaire, 2020).

Remote work has affected music improvisation practices. Despite modern remote communication technologies, remote work has had a detrimental impact on musicians' livelihood and practice. In response, new forms of social practices have begun to emerge, including increased use of elongated spaces and silences to facilitate remote music making sessions, new types of large-scale distributed music-making, and global, societal dialogues about music (Cai and Terry, 2020). Additionally, temporal lags due to network latency also created new opportunities to embrace the unexpected. These extreme circumstances have pushed musicians to listen more attentively to each other while playing together, a skill that is fundamental to music improvisation. Shelter-in-place has also given rise to new forms of collective experience such as global forums and projects for a greater cause. The global pandemic has motivated new strategies in collaborative music making, with emergent new forms of social creativity. These strategies for creating and sharing music under lockdown and shelter-in-place orders have been found to have diverse effects on wellbeing (Draper and Dingle, 2021) and connectedness among collaborating music-makers (Daffern et al., 2021) and between music-makers and their audiences (Onderdijk et al., 2021b).

Such a significant and abrupt change in musicians' environment, and these observations of how the extrinsic shock of the COVID-19 pandemic have impacted the musical community, raise several pressing questions. To what extent can livestreamed concerts and ubiquitous communities conduce feelings of social solidarity and resilience when physical gatherings are impossible? What new styles and genres of music arise from this collaborative technology heavy process? And how does expressivity transform from bodily expression to other forms of interaction?

In this study, our focus on musical collaboration unites existing literatures on collaborative creativity with a burgeoning field of research on the social and cultural psychology of music. Since the COVID-19 pandemic represents an unprecedented real-time event in both these strains of research, this study is a hybrid of inductive, exploratory methodologies and more directed, hypothesis-driven assessment. Such a mixed approach is ideally suited to both document a few of the ways in which the COVID-19 pandemic impacted the community of working musicians and to assess theories of music's functionality within social and cultural cognition.

We expected that musicians would most miss the loss of synchronicity in their interactions with fellow performers, since temporal alignment is crucial to phenomena such as musical entrainment and joint action which are themselves central to music-making (Renfrew et al., 2008; Phillips-Silver and Keller, 2012; Keller et al., 2014). We also expected that in attempting to maintain social ties within their respective musical communities, they would rely upon the connections furnished by their participation in specific musical genres and that the musical genres within which they choose to perform would adapt to changes in their collaborative networks; this prediction is driven by modern theories of musical genre which emphasize its connections to social, cultural, and technological affordances (Frith, 1996; Savage, 2006; Born, 2011; Brackett, 2016; Born et al., 2017; Born and Haworth, 2018). Furthermore, we expected that music-makers would draw upon nostalgic musical associations as sources of inspiration, both in their individual and collective musical practices, much like the effects Yeung found with music listeners (Yeung, 2020).

## 2. Overview of the Music-Making Process

There are myriad types of music-making, each with its own distinctive process, sociocultural function, and community. This variety has long been recognized as severely complicating any internally consistent definition of music and the process of its creation (Merriam, 1977; McClary, 1991; Bohlman, 1993). Furthermore, the Covid-19 pandemic impacted all of these communities differently, and it would be impossible to effectively summarize them all in one publication. In this paper, we focus on collaboration within communities of professional and semi-professional music makers in the United States operating in any musical style or genre. This focus was driven by the unique set of economic and social pressures the Covid-19 pandemic has exerted on the community of professional and semi-professional music makers, since they rely at least in part on making music for others for the financial and/or social security.

To effectively discuss the process of professional or semi-professional music-making, and define precisely what we mean by musical collaboration in that context, we must first sketch the general shape of this process. Although every instance is unique, this process can broadly be deconstructed into four phases, each of which involves a different set of roles. These phases are related to the “stages of music production” often cited by music bloggers (PQ, 2019), producers (Di Lorenzo et al., 2013), and music production instructors (Ottewill, 2020) but have been adapted to include the public or shared nature of the musical practices we are concerned with here. In addition, these stages frequently overlap, both in when they occur and who the participants at each point are, but this is a useful heuristic for conceptualizing music creation, especially in the context of collaboration.

The process begins with the writing or conceptualization phase. In this step, composers, songwriters, lyricists, arrangers, and their peers shape a piece of music. This phase involves writing a musical score in some genres (e.g., classical music), but a score is not expected or necessary in many other musical traditions. The nature of this shaping varies widely across musical cultures, as does the extent to which this phase is distinct from the others. For instance, this phase incorporates both of what Di Lorenzo and Zko call “composition” and “arrangement and instrumentation.” After a piece is conceptualized, it is generally performed. The performance step enlists musicians to realize the piece that was conceptualized in the prior step. In many traditions, the musicians also took part in the crafting of the piece, although some distinguish more strictly between the two roles.

If a piece is to be distributed beyond its immediate audience, it must be recorded in some fashion. At this point, recording engineers and producers join the creative process. Recording typically involves specific tasks such as mixing, or balancing the audio levels of different instruments, editing, or manipulating the sounds of individual tracks to achieve an aesthetic effect, and mastering, or manipulating the sound of the fully mixed and edited piece. Lastly, for a piece to be shared, it should be disseminated to some community of people. Close communities, such as friends and family, can receive shared music fairly easily, while disseminating music to larger communities often requires the use of technological resources such as social networks, and can require the assistance of publishers and publicists.

Individuals are not restricted to a single stage; in fact, many working music-makers play multiple roles at multiple steps in the creative process. In addition, the boundaries among these phases are extremely fuzzy. For example, composers frequently make changes to their pieces after hearing feedback from musicians, recording engineers, or music publishers.

In this context, collaboration is an interaction among at least two separate individuals, either within any layer or between any set of layers. Some of these interactions, such as those involving publicists and musicians, can be easily performed asynchronous, while others, most notably interactions among performers, face much steeper challenges. Network latency, or the time delay between an event happening on one end of a network connection and it being observed at the other end, has been shown to interfere with musicians' ability to effectively interact synchronously (Chafe et al., 2004), and asynchronous performance complicates attaining group flow states.

## 3. Survey

We employed two data collection approaches to assess the impact of Covid-19 on musical collaboration: a web-based survey and a set of one-on-one interviews. In this paper, we will present the methods and findings for the survey, followed by the methods and findings for the interviews, and will then discuss and interpret them in tandem. This hybrid methodology was approved by Stanford University's Institutional Review Board.

As the first stage in our data collection, we designed and deployed a survey using Qualtrics. This survey covered four basic topics: individual musical practice and inspirations, genre and style identification, collaboration network and behaviors, and the use of technologies such as production software and social media. Genre was assessed using a sliding scale for each of 14 genre categories, style was assessed using a 7-point scale for each of eight indicators, and musical inspiration was measured with 7-point scales for each of eight potential sources. Questions about internet usage and the usefulness of various forms of technology were also assessed using 7-point scales. A full list of questions is available in Appendix A. Participants indicated their consent by checking a box in the survey, as approved by Stanford University's IRB.

In order to balance the inductive, exploratory aspects of this research with its core hypotheses regarding the effects of the Covid-19 pandemic on musical practice, the questions for the survey were designed to provide a wide range of information concerning participants' musical practice before the pandemic, during the early stages of the pandemic, and during the most recent month before the survey was taken. The resulting data set could then be used both to directly assess the stated hypotheses and to implement exploratory methods for discovering relationships among the various musical style components, genres, and inspiration sources that are revealed by the extrinsic stress of the Covid-19 pandemic.

### 3.1. Methods

#### 3.1.1. Participants

**Figure 1:**
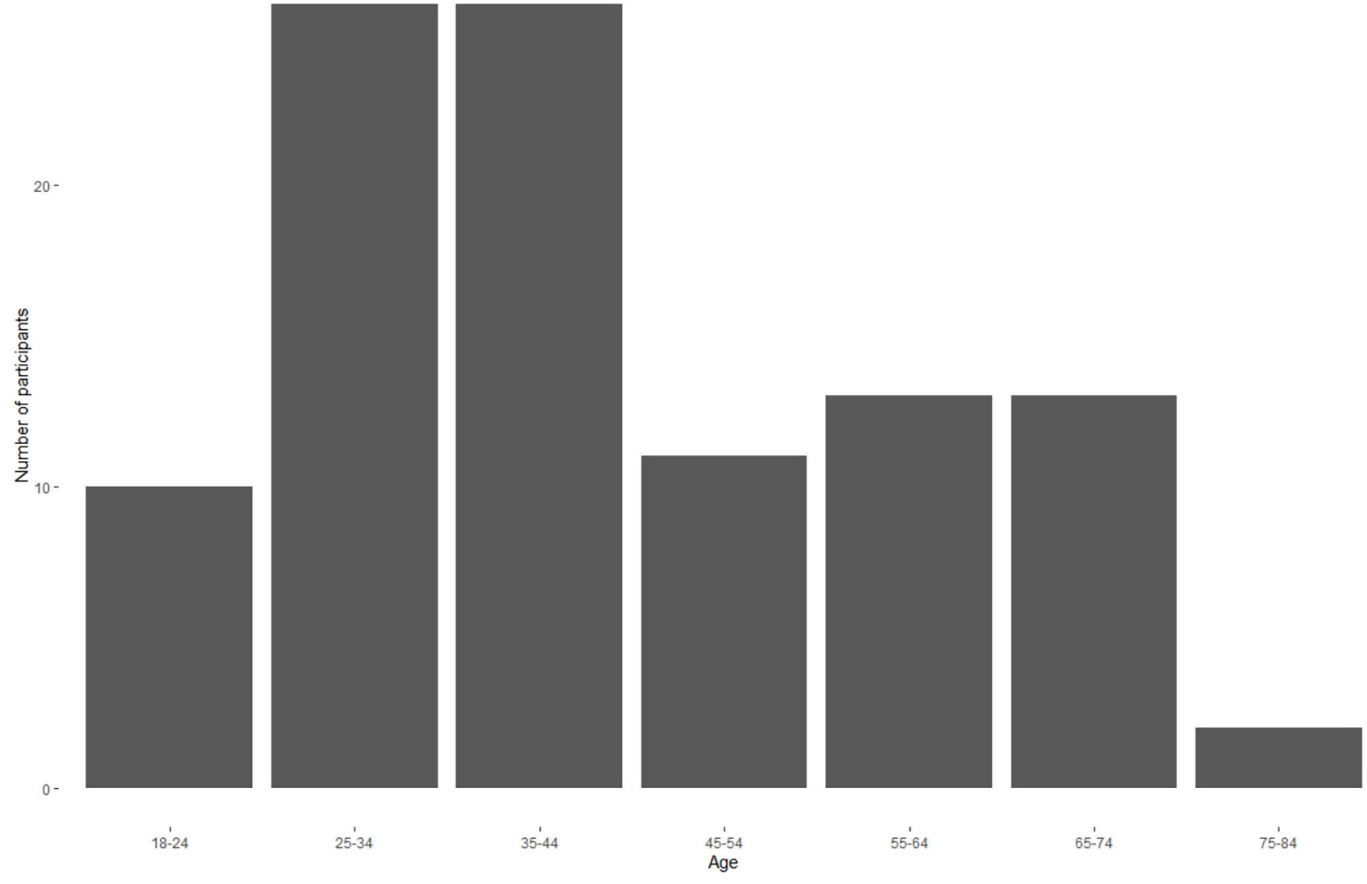
Histogram of participant ages.

Survey participants (N = 101, 65 male, 34 female, 2 other) were drawn from four communities of music creators: Young Entertainment Professionals, a Facebook community primarily located in Nashville, TN; the Grammy Museum; the Audio Engineering Society (AES); and the American Society of Composers, Authors, and Publishers (ASCAP). Participants were primarily clustered in California (14) and the tri-state area (20); there were 4 participants each in Tennessee, Illinois, and Quebec, and no other state or province had more than 3 participants. We did not collect data on ethnicity or cultural identification, and although we asked participants for their general age range, indicated in 5-year increments, we did not ask them to report their exact age. Figure 1 shows the distribution of participant ages.

#### 3.1.2. Survey Data Analysis

The quantitative survey data were analyzed using a combination of multivariate regression and exploratory factor analysis. Multiple linear regression models were built to predict each measure of musical style, inspiration, genre, and internet and social media usage using age, career length, time since shelter-in-place was first instituted, current state of shelter-in-place policy, phase of the pandemic (before shelter-in-place, during the first month of shelter-in-place, and during the most recent month), and interactions between age and career length and between time since SiP was first instituted and the current state of SiP policy. This analysis was aimed at assessing how music-makers' practice, including responses to survey items associated with musical collaboration, changed as a result of the pandemic. In data tables, we refer to time since shelter-in-place was first instituted as TSinceSiP, the current state of shelter-in-place policy as IsSiP, and the phase of the pandemic as SiPPhase.

As subjects were not required to respond to every question in the survey, some data were missing. Therefore, we restricted our analysis to participants who responded to at least 10% of the questions in the survey. This is a fairly permissive cutoff, since even after applying it, a majority of the remaining participants had not answered every question, but it resulted in a sufficiently dense data set for the linear models to be well-defined.

After the linear regression models were built using the initial dataset, missing data were estimated using multiple imputation, implemented using the MICE package in R (van Buuren and Groothuis-Oudshoorn, 2011). Exploratory latent factor analysis was then employed to search for relationships among all variables used in the regression analysis. Since this analysis was performed on a set of imputed datasets, this was implemented using pooled factor analysis functions in the psych package in R (Revelle, 2020). This factor analysis was intended to find relationships among survey responses related to elements of musical practice and reveal connections between measures of musical collaboration and of cultural resources such as musical genres, social resources such as existing networks of collaborators, and technological resources such as social media.

### 3.2. Survey Results

Means and standard deviations for variables related to collaboration and internet use, grouped by the presence of an active shelter-in-place order and the stage of the pandemic, are shown in Tables 1, 2, respectively. The linear regressions indicated several significant relationships between the variables measuring aspects of the pandemic – the presence of an active shelter-in-place order, the time since a shelter-in-place order was first instituted, and the stage of the pandemic – and several indicators of participants' musical practice. These results are summarized in Table 3. Here and in Table 3, β refers to the estimated coefficient of the linear relationship between the two variables. In the case of categorical variables such as IsSiP and SiPPhase, the coefficient refers to the effect of that variable taking on each value rather than a baseline value corresponding to a neutral state without pandemic-related restrictions. Throughout this section, *p*-values are presented without a correction in order to highlight plausible but marginal effects and maintain a consistent statistical threshold for all parts of the analysis.

**Table 1:** Mean and standard deviation for collaboration variables by shelter-in-place and stage of the pandemic.

	**IsSiP**		**SiPPhase**		
**Measure**	**Yes**	**No**	**Before**	**Early**	**Recent**
Style: Collaborative	3.56 (1.62)	3.85 (1.99)	4.06 (1.82)	3.09 (1.82)	4.21 (1.92)
Collab: Initiative	4.56 (2.12)	4.73 (2.11)	5.10 (1.96)	3.82 (2.00)	5.13 (2.13)
Collab: New collaborators	3.89 (1.91)	3.94 (1.84)	4.20 (1.72)	3.32 (1.80)	4.27 (1.91)
Collab: Mutual collaborators	3.77 (1.69)	4.29 (2.13)	4.39 (2.11)	3.88 (2.04)	4.25 (2.02)
Collab: Time difference problem	6.05 (0.85)	4.02 (1.89)	4.82 (1.89)	4.57 (1.83)	4.38 (2.02)

*These variables are Likert scales and are treated as continuous for this computation*.

**Table 2:** Mean and standard deviation for internet use by shelter-in-place and stage of the pandemic.

	**IsSiP**		**SiPPhase**		
**Measure**	**Yes**	**No**	**Before**	**Early**	**Recent**
Internet use: General	5.57 (1.72)	5.96 (1.35)	5.70 (1.29)	5.87 (1.72)	6.10 (1.23)
Internet use: Communication	5.67 (1.62)	5.06 (1.53)	5.00 (1.47)	5.13 (1.77)	5.38 (1.43)
Internet use: Music consumption	3.67 (1.28)	4.35 (1.79)	4.18 (1.65)	4.10 (1.87)	4.42 (1.69)
Internet use: Music creation	4.63 (1.54)	3.14 (1.98)	3.00 (1.87)	3.32 (2.04)	3.86 (2.02)

*These variables are ordinal with clearly-defined scalar relationships between responses, so are treated as continuous for this analysis*.

**Table 3:** Correlation matrix for pandemic-related predictors.

			**SiPPhase**	
**Measure**	**IsSiP**	**TSinceSiP**	**Early**	**Recent**
Time making music: Live			–3.766^***^	–2.347^*^
Time making music: Online			2.612^*^	3.312^**^
Internet use: Communication	4.008^***^			
Internet use: Music consumption		2.621^*^		
Internet use: Music creation	2.889^**^			2.589^*^
SM Usefulness: Collaboration		2.892^**^		
Style: Collaborative			–2.261^*^	
Genre: Blues	–2.060^*^			
Genre: Pop	–2.081^*^	–2.069^*^		
Inspiration: Nature	2.300^*^	2.146^*^		
Inspiration: Nostalgia	4.644^***^	3.205^**^		
Inspiration: Personal life		2.879^**^		
Inspiration: Social issues	3.307^**^			
Collab: Initiative			–3.045^**^	
Collab: New collaborators			–2.561^*^	
Collab: Time difference problem	8.281^***^	–4.760^***^		

*Only coefficients with significant p-values are shown (*

* *p < 0.05*,

** *p < 0.01, and*

*** *p < 0.001)*.

The early period in the pandemic had a strongly negative effect on time spent making music live (β = −3.766, *p* < 0.001) and a positive effect on time spent making music online (β = 2.612, *p* < 0.05). Similarly, the most recent month of the pandemic (as of survey completion) entailed significantly less time making music live (β = −2.347, *p* < 0.05) and significantly more time making music online (β = 3.312, *p* < 0.01) than before the pandemic. Participants spent more time using the internet for communication (β = 4.008, *p* < 0.001) and music creation (β = 2.889, *p* < 0.01) when under a shelter-in-place order than when not. In addition, the longer it had been since shelter-in-place was first instituted, the more time participants spent using the internet for music consumption (β = 2.621, *p* < 0.05) and the more useful they found social media for collaboration (β = 2.892, *p* < 0.01). The most recent month of the pandemic (as of survey completion) was associated with an increase in the time spent using the internet for music creation (β = 2.589, *p* < 0.05).

During the first month of the pandemic, participants indicated that the extent to which their music-making process was collaborative rather than solitary decreased (β = −2.261, *p* < 0.05). Being under a shelter-in-place order reduced the extent to which participants described their music as being Blues (β = −2.060, *p* < 0.05) and Pop (β = −2.081, *p* < 0.05), while the longer it had been since shelter-in-place was first instituted, the less likely participants were to describe their music as Pop (β = −2.069, *p* < 0.05). Other genres did not have significant relationships with any of our predictor variables, even with the lack of a statistical correction.

**Figure 2:**
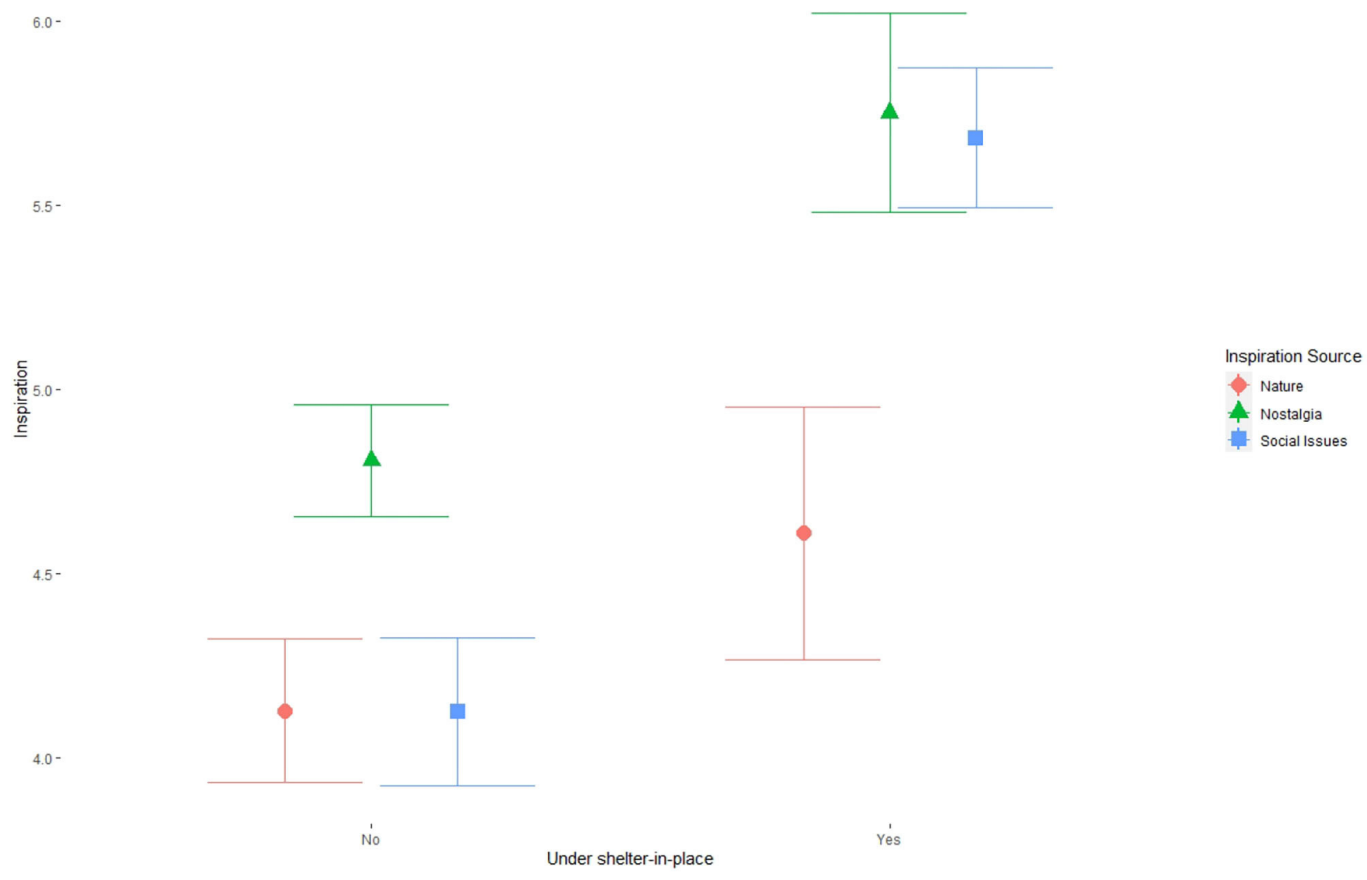
The effect of shelter-in-place orders on inspiration variables, showing mean and 95% confidence intervals. All variables were measured using 7-point Likert scales and lower values indicate a weaker fit between the variable and the music participants made.

Several sources of musical inspiration were impacted by shelter-in-place orders (Figure 2). Being under a shelter-in-place order when completing the survey led to significant increases in the extent to which participants drew on nature (β = 2.300, *p* < 0.05), nostalgia (β = 4.644, *p* < 0.001), and social issues (β = 3.307, *p* < 0.01). In addition, the longer it had been since shelter-in-place was first instituted in participants' area, the more likely they were to draw on nature (β = 2.146, *p* < 0.05), nostalgia (β = 3.205, *p* < 0.01), and their personal life (β = 2.879, *p* < 0.01) for musical inspiration. Of these sources of inspiration, nostalgia had the most consistent and dramatic increase in usage as a result of shelter-in-place orders.

**Figure 3:**
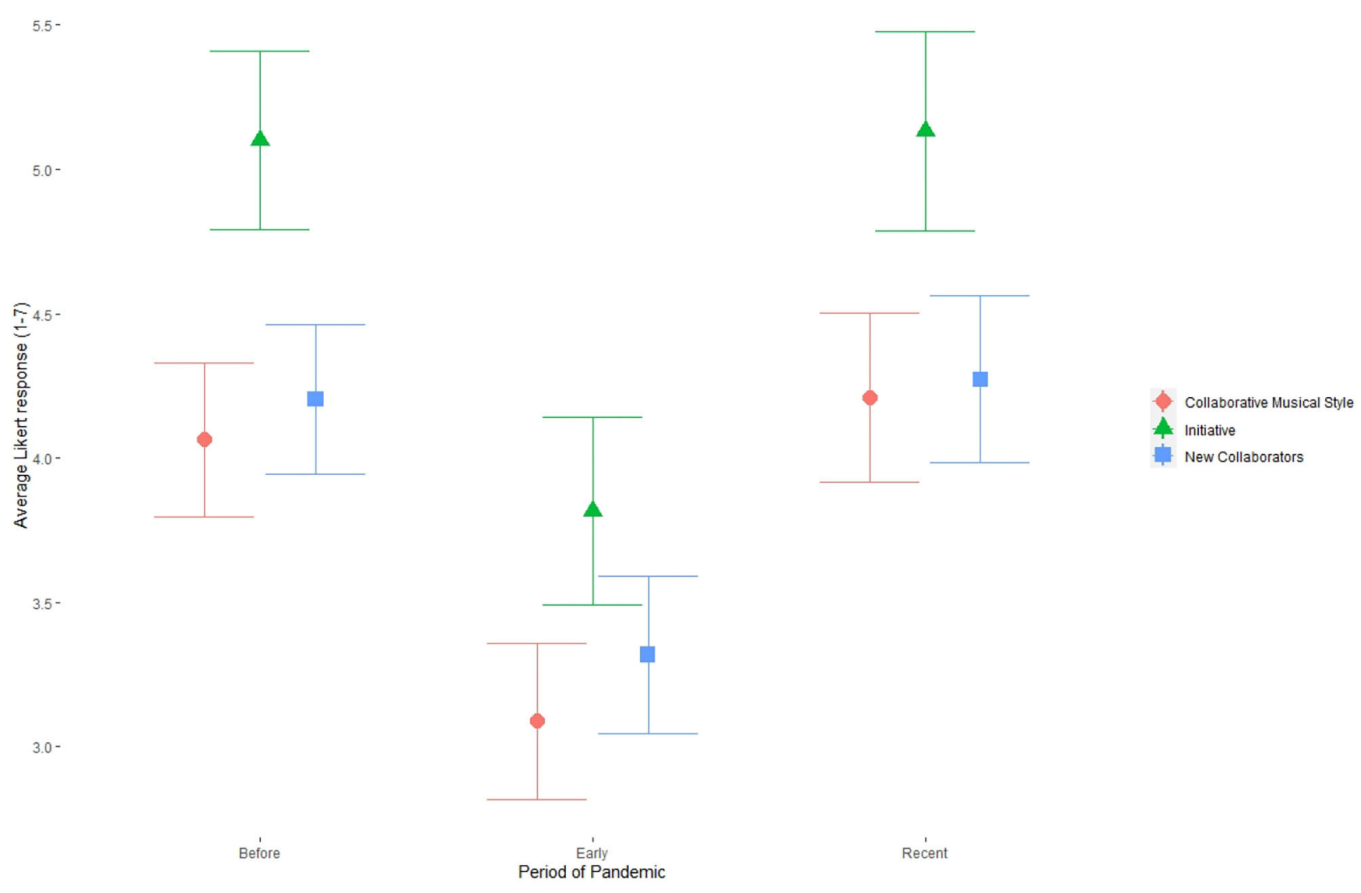
The effect of pandemic stage on collaboration variables, showing mean and 95% confidence intervals. Collaborative Musical Style measures how much participants work with others when making their music; Initiative measures how proactive participants are when seeking collaborators; and New Collaborators measures how likely participants are to collaborate with new people. All variables were measured using 7-point Likert scales and lower values indicate a weaker fit between the variable and the music participants made.

The difficulties posed by time zone differences were significantly increased when participants were under a shelter-in-place order (β = 8.281, *p* < 0.001), but were significantly ameliorated by more time since shelter-in-place was first instituted (β = −4.760, *p* < 0.001). The first month in the pandemic saw significant decreases in participants' initiative with seeking out collaborations (β = −3.045, *p* < 0.01) and their likelihood to seek new collaborators (β = −2.561, *p* < 0.05). The effect of the phase of the pandemic on variables related to collaboration is shown in Figure 3.

**Figure 4:**
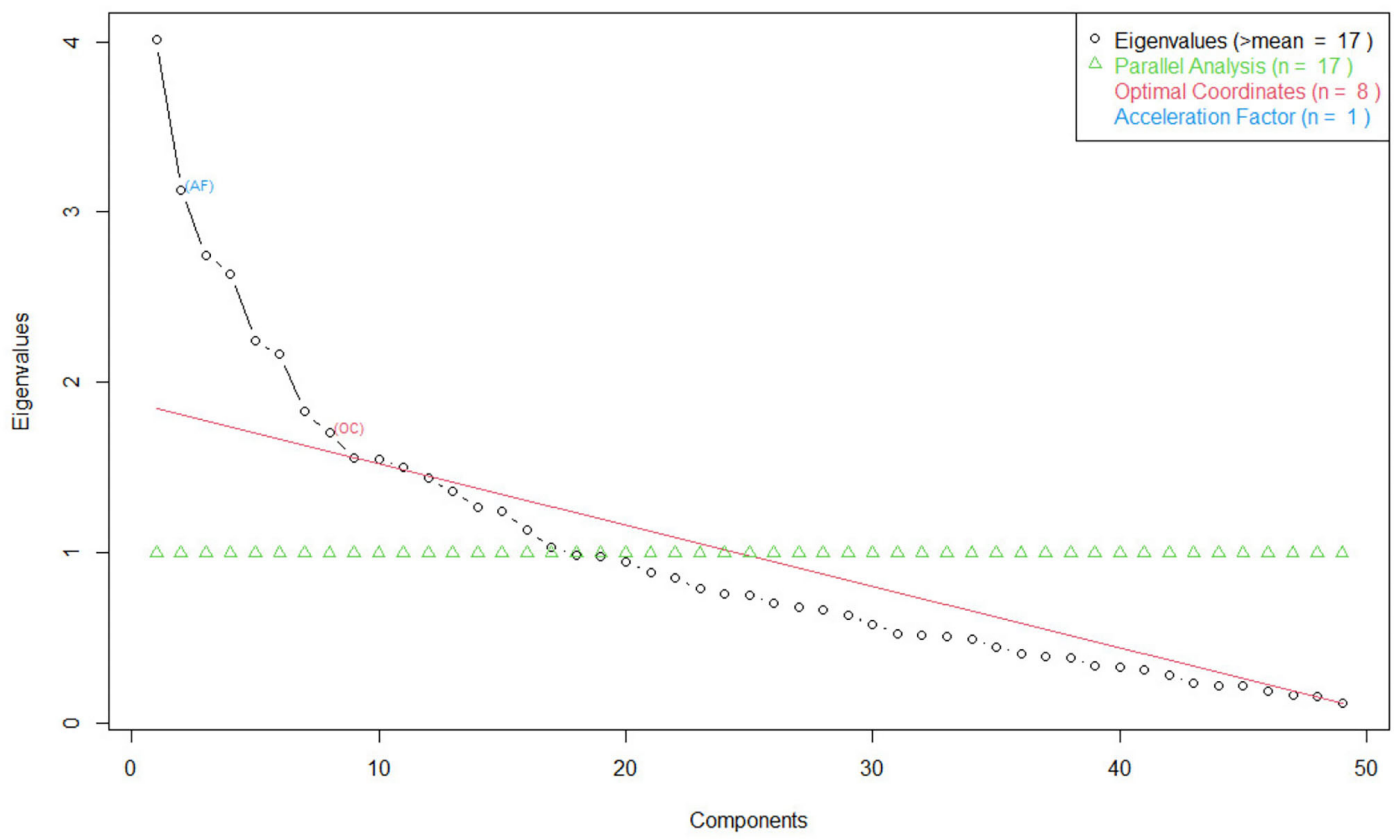
Scree plot of correlation matrix eigenvalues. Potential solutions are shown for parallel analysis (the point where the eigenvalues dip below 1), optimal coordinates (the first eigenvalue that lies above the line connecting the next eigenvalue and the last eigenvalue), and acceleration factor (the point where the curve's slope changes most sharply).

Due to the frequency with which participants answered portions of the survey, multiple imputation through predictive means matching was employed to fill gaps in the dataset to conduct an exploratory latent factor analysis. As this is an exploratory method, we did not determine the specific indicators which would load onto individual factors ahead of time, and determined the number of factors from an optimal coordinates analysis of the eigenvalues of the correlation matrix (Figure 4). A promax oblique rotation was employed in the factor analysis to account for possible colinearities among the resulting factors. Loadings with magnitude >0.3 onto the resulting eight factors are shown in Table 4. A summary of the interpretations of each factor is in Table 5.

**Table 4:** Factor loadings.

	**Factors**							
**Indicator**	**1**	**2**	**3**	**4**	**5**	**6**	**7**	**8**
Genre: Pop	0.85							
Genre: Folk	0.61							
SM Usefulness: General	–0.57							
Collab: Different time zones	–0.52							
SM Usefulness: Collab	–0.43							
Genre: Rock	0.43	–0.41						
Genre: Blues	0.41				0.33	–0.33		
Genre: Electronic	0.35	0.32						
Inspiration: Social issues	0.41	0.74						
Inspiration: Politics		0.71						–0.36
Inspiration: Other people's lives		0.7						
Inspiration: Literature		0.56						
Genre: Country		–0.4			0.31			
Internet Use: Music creation		0.36						0.36
Style: Electric			0.67					
Style: Loudness			0.59					
Style: Liveness			0.58					
Style: Originality			–0.49					
Inspiration: Other			0.48					
Genre: Religious			–0.47					
Inspiration: Religion			–0.4					
Genre: Alternative			0.34					
Collab: New collaborators				0.81				
Collab: Initiative				0.64				
Collab: Same Style				0.43				
Internet Use: Communication					0.68			
Internet Use: Any					0.66			
Internet Use: Music Consumption					0.46			
Genre: Other					0.38			
Inspiration: Nostalgia						0.63		
Genre: Metal						–0.51		
Inspiration: Personal Life						0.49	0.34	
Inspiration: Nature						0.48	–0.31	
Style: Collaborative				0.42		–0.44		
Inspiration: Science						0.4		
Style: Length						–0.35		
Genre: Hip-Hop							0.69	
Genre: Soundtrack							0.58	
Genre: Classical							0.54	
Style: Speed							–0.43	
Genre: Soul							0.35	
Style: Happiness							0.34	
Time Making music: Solo								0.55
Time Making music: Live								0.36
Genre: Jazz								0.31

*A promax oblique rotation was applied in obtaining these loadings to account for possible colinearities among the factors. Only loadings with magnitude greater than 0.3 are shown*.

**Table 5:** Interpretive labels for each factor.

**Factor**	**Label**
1	Poppiness
2	Activist musicking
3	Secular stadium
4	Musical extoversion
5	Internet usage
6	Musical introversion
7	Theatrical and cinematic musicking
8	Time making music

Interpreting the results of this analysis is necessarily subjective due to the strongly inductive nature of this analysis. Given our focus on musical collaboration, Factor 4 is of particular interest, especially when seen in the context of the correlations and interview responses. Since its sole indicators are the rate of new collaborators, initiative in seeking collaboration, a collaborative musical style, and a propensity to collaborate with other artists in the same style or genre, it likely represents a collaborative instinct, which we will call *musical extroversion*.

Nostalgia is the primary indicator loading onto Factor 6, along with the use of the artist's personal life, nature, or science as inspirations. Factor 6 is counter-indicated by the metal genre, a collaborative musical style, and creating long songs. This collection of features implies that Factor 6 may represent a tendency toward contemplative, internally focused music-making and away from loud, aggressive musical styles typical of genres such as metal; we will call this factor *musical introversion*.

Factor 5 is primarily indicated by three measures of internet use: time spent using the internet for communication, music consumption, and for any purpose at all, implying that it represents a general measure of *internet usage*. However, it is also weakly indicated by the blues and country genres, as well as indicating a genre not explicitly listed in the survey. The inclusion of these genres in this factor cannot be directly assessed based solely on the quantitative data available here. Similarly, while it is unsurprising that the amount of time spent making music on one's own and live, respectively, are both indicators of Factor 8, which we call *time making music*, their association with the jazz genre seems harder to explain. In both cases, information from the interview portion of this study may assist in interpreting these factors.

The remaining factors appear to represent clusters of genres or inspirations. Factor 1 shows a group of pop-adjacent genres with strong stylistic ties, implying it represents *poppiness*. The negative loadings connecting the usefulness of social media to this genre cluster may indicate a general attitude held by artists in that broad musical domain. Factor 2 is primarily indicated by a cluster of inspiration variables: social issues, politics, other people's lives, and literature. Therefore, this factor can likely be interpreted as a focus on external sources for songwriting inspiration, which can be called *activist musicking*. Interestingly, the country and rock genres both load negatively onto this factor, which may reflect the often apolitical basis of many contemporary country and rock songs, especially given country's strong associations with personal storytelling (Fox, 2004), arguments complicating rock's image of anti-commercialist authenticity (Frith, 1987; Attias, 2016), and both genres' shift, within the United States' musical zeitgeist, into a broader musical mainstream over the past half-century (Moore, 2005; Pecknold, 2007; see Hill, 2019; Ghlionn, 2021 for more polemical discussions of this phenomenon). Such genre shifts are increasingly situated as central to theories of genre, especially those that draw on anthropological or sociological sources (e.g., Brackett, 2016). This factor is also indicated, albeit mildly, by the use of the internet for music creation and by the “electronic” musical genre. Meanwhile, Factor 3 is dominated by three style-related indicators—electricness, loudness, and liveness—with smaller loadings for inspirations not listed in the survey and the “alternative” genre, and is also counter-indicated by originality and musical religiosity. This constellation of loadings may indicate a “Loud, Live, and Amplified” aesthetic, or a kind of *secular stadium* musical style. Factor 7's odd combination of hip-hop, soundtrack, and classical genres may be a consequence of changes in how music is used in stage works, films, or television placements, reflecting a general stylistic convergence of several musical cultures around theatrical and cinematic musicking.

## 4. In-Person Interviews

In order to reach a more nuanced understanding of the survey data, we conducted a series of Zoom interviews with willing participants. These interviews were semi-structured and touched on the same topics covered in the survey. The interviewer used a set of topics and suggested question wordings but conducted the interviews in as fluid and conversational a manner as possible while ensuring that each participant addressed each topic. A list of the interview topics and questions is in Appendix B.

Participants for the interviews were recruited from the pool of survey participants using a separate, optional form to ensure that potentially identifiable information was isolated from the survey data set. At the beginning of each interview, the interviewer read through the interview information sheet by sharing their screen with the participant and obtained verbal consent to proceed with the interview. Interviews took between 30 and 60 min and were recorded using OBS Studio; these recordings will be preserved for 3 years after completion of the study, as required by the IRB, but will not be made public to preserve the participants' privacy.

### 4.1. Methods

#### 4.1.1. Participants

Twenty individuals indicated their interest in being interviewed when they filled out the initial survey. Of those 20, 12 took part in interviews. Eight described their geographic environment as urban, three as suburban, and two as a town; one participant moved from a suburban environment to an urban one during the pandemic. In addition, while most interview participants (9) were long-term music creators, some indicated they were either restarting after a hiatus (3) or were transitioning to a new aspect of music-making (3), such as moving from music publicity to music recording. Interview participants were not asked about their gender identity, age, or ethnic or cultural background.

#### 4.1.2. Data Collection and Analysis

The interviews were annotated in Nvivo (QSR International Pty Ltd, 2020) using the constant comparative method of inductive coding (Glaser, 1965). Table 6 shows the broadest levels of code aggregation after application of the constant comparative method.

**Table 6:** Categories and codes for interview analysis.

**Category**	**Example codes**
Artistic opportunity	Open to taking risks
	Intimacy of virtual relationships
	Hunger for music and art
Internal focus	Solitary working practice
	Maintain momentum
	Nostalgia
Outward reach	Synergy of collaboration
	Maintain connections
	Shrunk the world
Social constraint	Real-time musical interaction
	Difficult multimedia collaboration
	Loss of boomer-age audience
Technical or structural constraint	Financial instability
	Music as content rather than art
	Own lack of technical knowledge
Ethical concerns	Community solidarity
	Not monetize COVID
	George Floyd
Internet use	Social media
	File sharing
	Remote DAW plugins (e.g., ListenTo, VSTConnect)
Standalone software use	DAWs (e.g., Logic, Pro Tools)
	Notation software (e.g., Sibelius, Finale)
	Virtual reality

### 4.2. Results

Every participant who discussed the severity of their personal response to Covid-19 described a careful and thorough response, involving rigorous mask-wearing and social distancing, as little activity outside the home or travel as possible, and careful hygiene. Older or at-risk participants expressed more caution, with one saying they “[had] not left [their] house for a year,” while younger participants were more flexible, with one saying “I go to the grocery store now, I will take the subway when I have to go to a doctor's appointment or something like that. I've eaten outside a few times, but...I wouldn't eat indoors, I wouldn't go to a friend's house, I wouldn't go to family, I wouldn't travel on Amtrak, I sure as fuck wouldn't fly.” Most of the participants described a serious response to Covid-19 within their community that fluctuated with the local severity of the pandemic; those that did not expressed concern over lax mask-wearing or said it was “quite shocking that the restaurants are open and people are in them and waiting in line, standing shoulder-to-shoulder.”

Only one participant indicated they had not been sheltering in place, with or without the existence of a formal shelter-in-place order. Three interview participants had contracted Covid-19 themselves, although all had recovered by the time of the interview. Almost every interview participant knew someone who contracted Covid-19, and six interview participants knew someone who had died from Covid-19. Four participants indicated their work had moved entirely online, while one person said they had a hybrid remote and in-person work situation, one said they were solely working in-person (albeit with health and safety precautions), and one was unemployed during the pandemic.

Table 6 shows the relationships among codes used in the analysis of the interview results that are directly relevant to the questions of collaboration and the use of social, cultural, or technical affordances. There were five top-level codes associated directly with participants' musical practice: *artistic opportunity, internal focus, outward reach, social constraint*, and *technical or structural constraint*. Some participants raised questions of *ethical concerns*, which are worth mentioning separately because of their direct connections with the broader context surrounding the COVID-19 pandemic. In addition, there were two primary classes of technology use participants cited: *internet use* and *standalone software use*; one participant also cited *physical apparatus use*, but most participants did not.

The prominence of *artistic opportunity* reflects the trend that although most participants indicated they had felt negative emotions during the pandemic, especially high-arousal negative emotions such as “desperation,” “existential anxiety,” and “panic,” participants showed a preference for discussing positive changes or silver linings of their pandemic experience. These ranged from realizations about their preferred creative and collaborative process to excitement at embracing new techniques and technological innovations for producing and sharing musical content. A music producer said they “have never gotten better and more focused notes and feedback that are more musically relevant,” while a singer expressed their enthusiastic embrace of the affordances of network technology by saying “there's just many amazing things happening now with technology, all accelerating because of the pandemic...you just have to think outside the box in how you implement them.” One participant praised what they perceived as the heightened intimacy of virtual relationships, saying “you're staring in each other's eyes all day long if you're on Zoom, and you can read every micro-gesture if you're really looking.” Some described how the virtualization of human interaction has allowed for contact across large distances.

Most participants indicated they had embarked on new collaborations during the pandemic, and most indicated they had initiated collaborations, although in both cases, participants mentioned an initial period of collaborative stagnation followed by a deliberate attempt to reclaim some of their old collaborative practice. These responses tended to overlap, with most participants who initiated collaborations also starting new ones during the pandemic. Combined, these trends exemplify the *outward reach* class of response. Many participants providing these responses expressed a desire to maintain their connections with their peers, drawing energy from musical collaboration, or that their musical process was generally outgoing and “favored the synergy of collaboration.” Outward reach responses also incorporated an interest in community solidarity and the ways in which shelter-in-place has shrunk the perceived effect of geographical distance. This connects *outward reach* to participants' *ethical concerns*. For instance, one participant cited their practice of making music based on stories of marginalized peoples, another expressed their unwillingness to write songs about COVID-19 due to their discomfort with monetizing the pandemic, and another described stopping their well-received effort to record 1-min videos on social media “when George Floyd was killed because it just felt...any bandwidth on anything but that felt very tone-deaf.”

*Social constraint* responses, on the other hand, were more concerned with the barriers shelter-in-place erected between potential collaborators. The most commonly cited barriers were concerned with the lack of real-time musical interaction, including live performance and playing in groups. Some mourned the separation of the creative community and worried that those effects might linger after the pandemic had ended, saying they “don't want us all to forget each other,” but that “the sad thing is that, like, I just don't know when I'll see [my collaborative partner] again.” Others highlighted perceptions of increased pushiness among potential collaborators, challenges facing multimedia collaborations, a lack of technical knowledge in potential or current collaborators, or shifts in the market for musical performance.

These responses overlapped with those indicating an *internal focus* in their music-making process, such as participants viewing themselves as the driving creative force in all their work or seeking internal sources for motivation such as perfectionism or maintaining creative momentum. *Internal focus* responses often carried a positive valence. In general, when participants expressed a specific opinion on a pandemic-induced change in their musical practice, they were more likely to regard changes in their personal creative process as positive and changes in their collaborative process as negative. Interview participants who felt the pandemic had aided their collaboration cited a preference for asynchronous collaborative work and a generally solitary creative practice. One such participant, describing their ideal collaborative process, said they “can complete a whole track and send it over… and then they get all the vocals immediately and just send it back.” The *internal focus* class of response also includes several emotional motivations, including references both to nostalgia. While many participants did not mention nostalgic sources, and several instead emphasized their excitement about the future of music-making, one explicitly described a friend and collaborator departing from their normal musical style and turning to their roots in the American South for musical inspiration, while another described an ongoing project writing an opera about their own life.

*Technical or structural constraint* responses emphasized phenomena such as the lack of economic stability, shift to virtual performances or teaching, and commodification of the music industry. They also dealt with the difficulties associated with specific technologies and participants' own lack of expertise in their use, especially network-based platforms for commissioning, creating, and sharing music. This was true across both *internet use*, including social networks, file sharing, messaging, livestream platforms, and remote audio tools, and *standalone software use*, including digital audio workstations (DAWs), virtual reality (VR), and notation software. In each case, participants cited both benefits and burdens to their musical collaboration associated with their increased reliance on these technologies.

## 5. Discussion

We set out to discover what aspects of musical collaboration music-makers strove to preserve and maintain and what social, cultural, and technological affordances they relied upon to do so. The quantitative results from the survey, which were left without corrections for multiple comparisons to include plausible but marginal effects, and qualitative findings from the interviews, when combined, offer compelling answers to both our core questions.

We believed that collaboration may have changed substantially because the Covid-19 pandemic directly interfered with the structures that normally enable collaboration, particularly in-person group congregant settings. This imposed a significant barrier to continuing collaborating as usual, which was borne out by the drop in collaborative behaviors during the first month of the pandemic. However, our finding that music-makers' collaborative practice rebounded during the pandemic itself implies that musicians are willing to commit a substantial quantity of resources to preserving their normal collaboration habits; this effect also appeared in the interviews, where participants consistently described the investments they and others in their artistic communities made to continue making music with each other, rather than just by themselves. Without the pandemic, and the specific obstacles it poses for interpersonal collaboration, especially for something as reliant on real-time, in-person interaction as music-making, the result that musicians tend to keep collaborating in the same way would not be terribly surprising. However, the pandemic-induced difficulty involved in maintaining the same collaborative behavior, coupled with the initial drop in collaboration overall, mean that this result carries strong implications about music-makers' priorities and the social role of music-making itself.

These implications are made more apparent by interpreting the interview data in the context of the factor analysis from the survey, particularly the *musical extroversion* factor. The existence of such a factor is borne out by the interviews, where participants who were more likely to initiate collaborations were the same ones who started new collaborations during the pandemic. In general, the pandemic solidified the collaborative approach of interview participants; those who were already inclined toward collaborative music-making continued to engage in collaborative practice, while those more invested in a solitary musical practice simply kept making music on their own.

The relationship between collaborating with other artists in the same style and *musical extroversion* also implies that the social and communal structures afforded by participation or membership in a musical genre are central to the collaborative approaches of music-makers. Since interview subjects indicated a preference for collaborating with friends or people with friendly attitudes, and the cases where a participant described venturing into a new musical genre were instigated by a close personal relationship, it seems likely that the connection between musical genre and collaboration is, at least in part, social rather than stylistic. This conclusion is supported by extant evidence for music's social efficacy (Hargreaves and North, 1999; Cross, 2001; Rabinowitch et al., 2013) and recent theorizations of musical genre that emphasize its social and cultural components (Born, 2011; Brackett, 2016).

Interview participants who thrived on collaboration cited an initial period of musical stagnation or the cessation of creative activity, followed by a deliberate attempt to recover something approaching their old collaborative musical practice. This trend is clearly apparent in the quantitative data as well (Figure 3), where the extent to which participants described their music as collaborative, their initiative in seeking collaborators, and the rate at which they worked with new people all declined during the first month of shelter-in-place orders, only to rebound by the time participants took the survey, which was still during the pandemic.

Taken together with the loading pattern onto the *musical extroversion* factor, these results would imply that music creators have a collaborative comfort zone that is remarkably resilient to even massive external shocks. If changes in music creators' environments force alterations in their collaborative pattern, they will seek ways to mimic their old collaborative habits, returning to a kind of creative homeostasis. It remains unclear how individuals would respond to significant extended or indefinite changes to their environments' collaborative affordances; several participants expressed worry over the future of their ongoing collaborations or doubt that they would be collaborating at all if their projects had not begun before the pandemic. However, given a reasonable expectation of structural stability in the long term, music creators will most often successfully restore their normal collaborative habits, indexed by *musical extroversion*, even in spite of massive short-term interference.

The significant effects of shelter-in-place orders on sources of musical inspiration (Figure 2), indicating clear increases in the extent to which survey participants drew on social issues and nostalgia while quarantined, may be partly explained by events not directly related to the pandemic—in fact, several interview participants directly referenced this possible confound. However, nostalgia's increased relevance is of a piece with Yeung's findings with respect to music consumption (Yeung, 2020). This represents a significant point of departure between the interviews and the survey. While the survey data show a strong impact of shelter-in-place orders on the use of nostalgia as inspiration, only two interview participants mentioned that either they or some of their colleagues had drawn more heavily than usual on their past in their music-making; one explicitly described a friend and collaborator turning to their roots in the American South, while another described an ongoing project writing an opera about her own life. In contrast, most interviewees demonstrated an interest in the future, whether that interest was tinged with excitement or existential dread. This indicates a potential distinction between different ways of coping with the stress of pandemic-induced isolation that may be connected to the construction of the interview sample itself. Opt-in interview strategies often result in selection biases. In this particular case, it is possible that the population that volunteered to be interviewed was simply more likely than the rest of the population to be future-oriented. Given the technologically mediated nature of the study and its dissemination, such a bias seems both eminently plausible and liable to be exacerbated by the stresses imposed by the Covid-19 pandemic.

The interview data also offers some potential insights into the internal structures of the *internet usage* and *time making music* factors. In the former instance, the small positive loadings of blues and country music onto *internet usage* may be connected to nostalgia sometimes associated with both genres. Alternatively, this may be connected to pre-existing internet-mediated subgenres such as alternative country (Peterson and Beal, 2001; Lee and Peterson, 2004). Either effect is worth further investigation, as there is currently insufficient evidence to fully justify either interpretation.

The fact that identifying as a jazz musician has a small positive loading onto *time making music* is difficult to explain concretely, but a quirk exposed by the interviews may offer a speculative rationale. While several participants indicated that they had a background in jazz or that their music was jazz-inspired, none of them were currently engaged in making jazz music. This might imply that jazz training and practice can be a route into earning gigs in a variety of genres, much as classical vocal training is often sought by singers outside the typical classical music realm. *Time making music* is also indicated by the use of the internet for music creation, and is counter-indicated by political inspirations for musical content. It may be that these connections are due to the necessity of internet use for music-making during the pandemic and the desire to make music that channels positive emotions or distracts from current events. One jazz-trained musician who was interviewed said they responded to political or social events during the pandemic by restricting their musical output so as not to take up space in the public sphere, another explicitly stated that they avoided working with political or social causes in their work as a commercial songwriter, and none of the other jazz-trained interview participants cited any political issue as a source of inspiration.

Interview data can also help unpack the internal structure of *poppiness*, particularly the strong negative loadings of social media usefulness in general and for collaboration. Several interview participants cited the genres loading onto *poppiness* as inspirations, especially rock-like and popular music genres, and those either did not mention social media specifically or viewed it as a necessary evil more than a particular boon for music-making. While the negative loading on the rate of collaboration across multiple time zones may imply that artists within this genre cluster operate mostly within their close geographic vicinity, this interpretation is neither supported nor contested by interview data, and remains purely speculative.

## 6. Conclusions

We found that music-makers' collaborative practices are fairly resilient to extrinsic shocks. After a period of depressed collaboration induced by the onset of the Covid-19 pandemic, both survey and interview data indicated that music-makers moved to restore their pre-pandemic approach to musical collaboration. In this process, music-makers tended to prioritize collaborative or communal aspects of music-making over other factors, even musical style. This aligns with our prediction that musicians would devote their energy to preserving social and communal aspects of their music-making process whenever possible. Interview participants indicated that they ventured into new stylistic terrain as a result of successful collaborations. This supports the underlying theory that social and cultural ties are as central to genres as similarities in the music itself, although many of our proposed relationships between genres and other indicators of musical practice are speculative in nature and require further research to fully validate. However, participants adjusted their collaborative practices to fit shifts in the available modalities of music-making and the genres and inspirations at the foundation of their musics. Music-makers' attitudes toward technological tools, in particular, remained relatively constant in the face of the pandemic, contributing to individual differences in how music-makers engaged with each other.

The Covid-19 pandemic and its associated shelter-in-place orders also instigated significant shifts in these sources of creative inspiration. Most notably, our hypothesis that music-makers would rely more heavily on nostalgia as a source of inspiration was supported by both the survey and interview results. Some of these shifts, such as the rise in inspiration from social issues, may be due to parallel events such as the protests sparked by the death of George Floyd. However, the increased relevance of nostalgic inspirations is likely a direct result of pandemic-induced isolation.

Our factor analysis offers some potential explanations of the forces underlying many of these effects. We found that personal and musical traits such as *musical extroversion, musical introversion*, and *activist musicking* appear to be at the core of the stability of music-makers' collaborative practice, the increased provenance of nostalgic musical inspirations, and the increase in socially and politically relevant music-making, respectively. The separability of these components indicates that they may be generalizable beyond the context of this pandemic, although confirmatory analyzes with expanded data sets are necessary to establish this relationship.

## Data Availability Statement

The raw data supporting the conclusions of this article will be made available by the authors, without undue reservation.

## Ethics Statement

The studies involving human participants were reviewed and approved by Administration Panel on Human Subjects in Medical Research, IRB 21, Stanford University. The patients/participants provided their written informed consent to participate in this study.

## Author Contributions

NF, VG, HT, and JB: survey, interview design, and writing. NF: data collection and analysis. All authors contributed to the article and approved the submitted version.

## Conflict of Interest

The authors declare that the research was conducted in the absence of any commercial or financial relationships that could be construed as a potential conflict of interest.
