# Supplementary material for: Collaborating in Isolation: Assessing the Effects of the Covid-19 Pandemic on Patterns of Collaborative Behavior Among Working Musicians
Source: Front Psychol. 2021 Jul 19;12:674246. doi: 10.3389/fpsyg.2021.674246 (PMC8326970; doi:10.3389/fpsyg.2021.674246)
Supplement: Supplementary file 1 [file Data_Sheet_1.pdf]

Appendices

A SURVEY QUESTIONS

Gender What is your gender? (Male, Female, Other, Prefer not to say)

Age How old are you?

- 18-24 years
- 25-34 years
- 35-44 years
- 45-54 years
- 55-64 years
- 65-74 years
- 75-84 years
- More than 85 years

NorthAmerica Do you live in North America (Canada, the United States, Mexico, and territories affiliated with those countries)? (Yes/No)

Location What country and state or province do you live in? (Dropdown)

Environment Which of the following best describes the kind of environment you live in?

- City
- Suburb
- Town
- Rural/countryside

TSinceSiP About how long has it been since shelter-in-place was first implemented in your area?

- Less than 1 month
- 1-3 months
- 3-6 months
- More than 6 months

IsSiP Are you currently under a shelter-in-place order? (Yes/No)

Creator Are you involved in creating music in any role, such as musician, writer/composer, engineer, or producer? Please select the one that best describes your status.

- No, I do not create music
- I create music, but I do not earn money for it
- I create music and earn money for it, but it is not my primary source of income
- I create music and earn money for it as my primary source of income

CareerLength If you earn money for your music, whether it is your primary source of income or not, how long have you earned money for your music?

- I do not earn money for my music
- 1 year or less
- 2-5 years
- 5-10 years
- More than 10 years

GenrePreCovid How much did these genres describe the music you made before the Covid-19 pandemic? (Not at all – 0; Somewhat – 50; Very well – 100)

- Alternative
- Blues
- Classical
- Country
- Electronic
- Folk
- Heavy metal
- Jazz
- Pop
- Hip-hop
- Religious
- Rock
- Soul
- Soundtrack
- Other (please specify)

GenreEarlySiP How much did these genres describe the music you made during the first month of shelter-in-place? (Not at all – 0; Somewhat – 50; Very well – 100)

- Alternative
- Blues
- Classical
- Country
- Electronic
- Folk
- Heavy metal
- Jazz
- Pop
- Hip-hop
- Religious
- Rock
- Soul

- Soundtrack
- Other (please specify)

GenreRecent How much did these genres describe the music you made during the past month? (Not at all – 0; Somewhat – 50; Very well – 100)

- Alternative
- Blues
- Classical
- Country
- Electronic
- Folk
- Heavy metal
- Jazz
- Pop
- Hip-hop
- Religious
- Rock
- Soul
- Soundtrack
- Other (please specify)

TSoloPreCovid Before the Covid-19 pandemic, about how many hours per week did you spend making music by yourself? (Number box)

TSoloEarlySiP During the first month of shelter-in-place, about how many hours per week did you spend making music by yourself? (Number box)

TSoloRecent During the past month, about how many hours per week did you spend making music by yourself? (Number box)

TLivePreCovid Before the Covid-19 pandemic, about how many hours per week did you spend making music with others in person? (Number box)

TLiveEarlySiP During the first month of shelter-in-place, about how many hours per week did you spend making music with others in person? (Number box)

TLiveRecent During the past month, about how many hours per week did you spend making music with others in person? (Number box)

TOnlinePreCovid Before the Covid-19 pandemic, about how many hours per week did you spend making music with others online? (Number box)

TOnlineEarlySiP During the first month of shelter-in-place, about how many hours per week did you spend making music with others online? (Number box)

TOnlineRecent During the past month, about how many hours per week did you spend making music with others online? (Number box)

StylePreCovid In general, the music I made before shelter-in-place was... (scale from 1 to 7)

- Quiet ↔ Loud
- Fast ↔ Slow
- Short ↔ Long
- Acoustic ↔ Electric
- Sad ↔ Happy
- Live ↔ Recorded
- Solo ↔ Collaborative
- Original ↔ Covers or Remixes

StyleEarlySiP In general, the music I made during the first month of shelter-in-place was... (scale from 1 to 7)

- Quiet ↔ Loud
- Fast ↔ Slow
- Short ↔ Long
- Acoustic ↔ Electric
- Sad ↔ Happy
- Live ↔ Recorded
- Solo ↔ Collaborative
- Original ↔ Covers or Remixes

StyleRecent In general, the music I have made during the past month was... (scale from 1 to 7)

- Quiet ↔ Loud
- Fast ↔ Slow
- Short ↔ Long
- Acoustic ↔ Electric
- Sad ↔ Happy
- Live ↔ Recorded
- Solo ↔ Collaborative
- Original ↔ Covers or Remixes

InspirePreCovid In general, the music I made before shelter-in-place was inspired by... (scale from 1 to 7)

- My personal life
- Politics
- Nostalgia
- Literature
- Other people's lives
- Nature

- Religion
- Social issues
- Science
- Other (please specify)

InspireEarlySiP In general, the music I made during the first month of shelter-in-place was inspired by... (scale from 1 to 7)

- My personal life
- Politics
- Nostalgia
- Literature
- Other people's lives
- Nature
- Religion
- Social issues
- Science
- Other (please specify)

InspireRecent In general, the music I made during the past month has been inspired by... (scale from 1 to 7)

- My personal life
- Politics
- Nostalgia
- Literature
- Other people's lives
- Nature
- Religion
- Social issues
- Science
- Other (please specify)

CollabSamePreCovid Before shelter-in-place, to what extent did you collaborate with people who make the same kind of music as you do?

- I do not make music
- I did not collaborate before shelter-in-place
- All of my collaborators made the same kind of music as me
- Most of my collaborators made the same kind of music as me
- Some of my collaborators made the same kind of music as me
- A few of my collaborators made the same kind of music as me
- None of my collaborators made the same kind of music as me

CollabSameEarlySiP During the first month of shelter-in-place, to what extent did you collaborate with people who make the same kind of music as you do?

- I do not make music
- I have not collaborated since shelter-in-place
- All of my collaborators made the same kind of music as me
- Most of my collaborators made the same kind of music as me
- Some of my collaborators made the same kind of music as me
- A few of my collaborators made the same kind of music as me
- None of my collaborators made the same kind of music as me

CollabSameRecent During the past month, to what extent have you collaborated with people who make the same kind of music as you do?

- I do not make music
- I have not collaborated during the past month
- All of my collaborators made the same kind of music as me
- Most of my collaborators made the same kind of music as me
- Some of my collaborators made the same kind of music as me
- A few of my collaborators made the same kind of music as me
- None of my collaborators made the same kind of music as me

CollabContEarlySiP During the first month of shelter-in-place, did you continue collaborating with the people you worked with before the pandemic?

- I do not make music
- I did not collaborate before shelter-in-place
- I continued collaborating with all of those people
- I continued collaborating with most of those people
- I continued collaborating with some of those people
- I continued collaborating with a few of those people
- I did not continue collaborating with any of those people

CollabContRecent During the past month, have you continued collaborating with the people you worked with before the pandemic?

- I do not make music
- I have not collaborated since shelter-in-place
- I continued collaborating with all of those people
- I continued collaborating with most of those people
- I continued collaborating with some of those people
- I continued collaborating with a few of those people
- I did not continue collaborating with any of those people

CollabContOnlinePre For people who you collaborated with before the pandemic and have continued collaborating with since, did you make music online together before shelter-in-place?

- I do not make music
- I did not collaborate before shelter-in-place
- I have not continued collaborating with anyone I worked with before shelter-in-place
- I frequently made music online with them
- I sometimes made music online with them
- I never made music online with them

CollabContOnlineNow For people who you collaborated with before the pandemic and have continued collaborating with since, do you make music online together now?

- I do not make music
- I did not collaborate before shelter-in-place
- I have not continued collaborating with anyone I worked with before shelter-in-place
- I frequently make music online with them
- I sometimes make music online with them
- I never make music online with them

CollabNContOnlinePre For people who you collaborated with before the pandemic and have not continued collaborating with since, did you make music online together before shelter-in-place?

- I do not make music
- I did not collaborate before shelter-in-place
- I have not continued collaborating with anyone I worked with before shelter-in-place
- I frequently made music online with them
- I sometimes made music online with them
- I never made music online with them

FCollabNew How frequently did you collaborate with new people (e.g. people you had never collaborated with before) during each time period? (scale from 1 to 7, with 1: never and 7: all the time)

- Before the Covid-19 pandemic
- During the first month of shelter-in-place
- During the last month

CollabMeetPreSiP If you collaborated with new people before the pandemic, how did you meet them? Please select all that apply.

- I do not make music
- I did not collaborate with new people
- We were introduced through friends
- We met over social media or some other online platform
- We met through a professional matching service

- We met by chance
- We knew each other before, but started to collaborate recently
- Other (with text box)

CollabMeetEarlySiP If you collaborated with new people during the first month of shelter-in-place, how did you meet them?

- I do not make music
- I did not collaborate with new people
- We were introduced through friends
- We met over social media or some other online platform
- We met through a professional matching service
- We met by chance
- We knew each other before, but started to collaborate recently
- Other (with text box)

CollabMeetRecent If you have been collaborating with new people during the past month, how did you meet them?

- I do not make music
- I have not been collaborating with new people
- We were introduced through friends
- We met over social media or some other online platform
- We met through a professional matching service
- We met by chance
- We knew each other before, but started to collaborate recently
- Other (with text box)

CollabEachOther About how many of the people you collaborated with during each time period collaborate with each other as well as with you? (scale from 1 to 7, with 1: none and 7: all) - Before the Covid-19 pandemic - During the first month of shelter-in-place - During the last month

Initiative To what extent did you initiate collaborations during each time period? (scale from 1 to 7, with 1: never and 7: all the time) - Before the Covid-19 pandemic - During the first month of shelter-in-place - During the last month

CollabOtherTPreSiP Before shelter-in-place, did you collaborate with people in different time zones? (Yes/No)

TDiffBarrierPre If you collaborated with people in different time zones before shelter-in-place, would you agree or disagree that the time difference was a barrier to collaboration or performance? (scale from 1 to 7, with 1: strongly disagree and 7: strongly agree)

CollabOtherTEarlySiP During the first month of shelter-in-place, did you collaborate with people in different time zones? (Yes/No)

TDiffBarrierEarly If you collaborated with people in different time zones during the first month of shelter-in-place, would you agree or disagree that the time difference was a barrier to collaboration or performance? (scale from 1 to 7, with 1: strongly disagree and 7: strongly agree)

CollabOtherTRecent During the past month, have you collaborated with people in different time zones? (Yes/No)

TDiffBarrierRecent If you have collaborated with people in different time zones during the past month, would you agree or disagree that the time difference has been a barrier to collaboration or performance? (scale from 1 to 7, with 1: strongly disagree and 7: strongly agree)

GeoRangePreSiP What was the geographic range of your network of collaborators before shelter-in-place?

- I do not make music
- I did not collaborate before shelter-in-place
- 30 minutes travel
- 1 hour travel
- 2 hours travel
- State
- Time zone
- Country
- Region (e.g. North America, South America, Asia, Europe, etc.)
- Global

GeoRangeEarlySiP What was the geographic range of your network of collaborators during the first month of shelter-in-place?

- I do not make music
- I have not collaborated since shelter-in-place
- 30 minutes travel
- 1 hour travel
- 2 hours travel
- State
- Time zone
- Country
- Region (e.g. North America, South America, Asia, Europe, etc.)
- Global

GeoRangeRecent What has the geographic range of your network of collaborators been during the past month?

- I do not make music
- I have not collaborated during the past month
- 30 minutes travel

- 1 hour travel
- 2 hours travel
- State
- Time zone
- Country
- Region (e.g. North America, South America, Asia, Europe, etc.)
- Global

HpWInternetAny About how many hours per week did you use the internet for any purpose during each period? (scale from 1-7, with 1:less than 1 hour and 7: more than 20 hours)

- Before the Covid-19 pandemic
- During the first month of shelter-in-place
- During the last month

HpWInternetCreate About how many hours per week did you use the internet to create music during each period? (scale from 1-7, with 1:less than 1 hour and 7: more than 20 hours)

- Before the Covid-19 pandemic
- During the first month of shelter-in-place
- During the last month

HpWInternetConsume About how many hours per week did you use the internet to listen to or consume music during each period? (scale from 1-7, with 1:less than 1 hour and 7: more than 20 hours)

- Before the Covid-19 pandemic
- During the first month of shelter-in-place
- During the last month

HpWInternetTalk About how many hours per week did you use the internet for interpersonal communication, whether related to music or not? (scale from 1-7, with 1:less than 1 hour and 7: more than 20 hours)

- Before the Covid-19 pandemic
- During the first month of shelter-in-place
- During the last month

CollabStrat When collaborating during shelter-in-place, what performance or recording strategies have you used?

- I did not make music during shelter-in-place
- I have not collaborated since shelter-in-place
- Playing together in real time online
- Overdubbing a track someone else recorded
- Playing to a metronome or click track and editing multiple tracks together
- Other (with text box)

LearnTechs Did you spend time learning new technologies to facilitate your music making at any point during shelter-in-place?

CollabTech Have you collaborated with music engineers and technology experts to facilitate your music making at any point during shelter-in-place? (Yes/No)

SMUseful How useful was social media when connecting with your listeners during each time period? (scale from 1-7, with 1: extremely useless and 7: extremely useful)

- Before the Covid-19 pandemic
- During the first month of shelter-in-place
- During the last month

SMPlatUsePreCov Before the Covid-19 pandemic, how useful was each of these social media platforms when connecting with your listeners? Please rank them from most useful (1) to least useful (9).

- Facebook
- Twitter
- Instagram
- TikTok
- Twitch
- YouTube
- Vimeo
- Snapchat
- Other (please specify)

SMPlatUseEarlyCov During the first month of shelter-in-place, how useful was each of these social media platforms when connecting with your listeners? Please rank them from most useful (1) to least useful (9).

- Facebook
- Twitter
- Instagram
- TikTok
- Twitch
- YouTube
- Vimeo
- Snapchat
- Other (please specify)

SMPlatUseLastM During the past month, how useful have each of these social media platforms been when connecting with your listeners? Please rank them from most useful (1) to least useful (9).

- Facebook
- Twitter
- Instagram

- TikTok
- Twitch
- YouTube
- Vimeo
- Snapchat
- Other (please specify)

SMUseCollab How useful was social media when finding and working with collaborators during each time period? (scale from 1-7, with 1: extremely useless and 7: extremely useful)

- Before the Covid-19 pandemic
- During the first month of shelter-in-place
- During the last month

SMPlatCollabPreCov Before the Covid-19 pandemic, how useful was each of these social media platforms when finding and working with collaborators? Please rank them from most useful (1) to least useful (9).

- Facebook
- Twitter
- Instagram
- TikTok
- Twitch
- YouTube
- Vimeo
- Snapchat
- Other (please specify)

SMPlatCollabEarlyCov During the first month of shelter-in-place, how useful was each of these social media platforms when finding and working with collaborators? Please rank them from most useful (1) to least useful (9).

- Facebook
- Twitter
- Instagram
- TikTok
- Twitch
- YouTube
- Vimeo
- Snapchat
- Other (please specify)

SMPlatCollabLastM During the past month, how useful have each of these social media platforms been when finding and working with collaborators? Please rank them from most useful (1) to least useful (9).

- Facebook
- Twitter
- Instagram
- TikTok
- Twitch
- YouTube
- Vimeo
- Snapchat
- Other (please specify)

SMUsefulFR What aspects of social media have you found most useful for you in your own musical practice? Why? (Free response)

SMNotUsefulFR What aspects of social media have you found least useful for you in your own musical practice? Why? (Free response)

SMUsefulGenreFR What social media platform have you found most useful for musical artists in your genre? Why? (Free response)

SMNotUsefulGenreFR What social media platform have you found least useful for musical artists in your genre? Why? (Free response)

SMGoodInteractFR What types of interactions (such as sharing new recordings, real-time audience responses in live performances, interpersonal communication, and listener feedback to music) with your listeners have translated the most effectively to social media? Why? (Free response)

SMBadInteractFR What types of interactions (such as sharing new recordings, real-time audience responses in live performances, interpersonal communication, and listener feedback to music) with your listeners have translated the least effectively to social media? Why? (Free response)

B INTERVIEW QUESTIONS

About how long have you been a music creator?

What country/city do you live in?

How would you describe your neighborhood? Is it rural, urban, suburban, or something else?

Are you currently under a shelter-in-place order, or have you been sheltering in place even without one?

How long has it been since your area first started requiring or recommending that people shelter in place? How consistent has that requirement or recommendation been since then?

What kinds of personal experiences, if any, have you had with Covid-19? Have you or anyone you know gotten it or been exposed to it?

I know a lot of musicians drawn on a lot of different styles, genres, and inspirations to make their music, and I'd like to know more about yours. There's been a lot of changes in music during the Covid-19 pandemic, so let's start back before the pandemic started. How would you describe the music you were making back then?

Once the pandemic started, how did the music you were making change?

How would you describe the music you've been making recently?

Did you collaborate with other music creators before the Covid-19 pandemic?

- If yes: How did those collaborations usually work?
- If no: Why not?

Once the pandemic started, how did your collaborations, and the people you collaborated with, change?

Have you been collaborating with other music creators recently?

- If yes: How have those collaborations usually worked?
- If no: Why not?

What kinds of technology, like recording software, social media, or audiovisual platforms like Zoom, Google Hangouts, or jacktrip? What did you find the most useful, and why?

How did the technology you use to make music, and how useful you found that technology, change once the Covid-19 pandemic started?

What kinds of technology have you been using recently?

How do you think the technology you've been using since the pandemic has fallen short in helping you make your music? How has it done well?

The Covid-19 pandemic has affected artists in a lot of ways, and I know I didn't ask specifically about all of them. What else has changed for you, as a music creator, since the pandemic started?
